# American Association of Obstetricians and Gynecologists

**Published:** 1899-11

**Authors:** 


### AMERICAN ASSOCIATION OF OBSTETRICIANS AND GYNECOLOGISTS.

(*Canadian Practitioner*, October.)

THE twelfth annual meeting of the American Association of Obstetricians and Gynecologists took place in the assembly room of the Denison House, Indianapolis, Ind., on Tuesday, Wednesday and Thursday, September 19, 20 and 21, 1899, under the presidency of Dr. Edward J. Ill, of Newark, New Jersey.

The program consisted of a short address by the Mayor, followed by a response of the President on behalf of the Association. Many very interesting papers were presented. Among the essayists were: Drs. J. F. Baldwin, of Columbus; L. H. Dunning, of Indianapolis; L. S. McMurtry, of Louisville; D. Tod Gilliam, of

Columbus; Robert T. Morris, of New York; Edwin Ricketts, of Cincinnati; W. H. Humiston, and M. Rosenwasser, of Cleveland; Walter B. Chase, of New York; Frederick Blume, of Allegheny; J. M. Duff, of Pittsburg; W. E. B. Davis, of Birmingham, Ala.; W. H. Myers, of Fort Wayne; Rufus B. Hall, of Cincinnati; J. F. W. Ross, of Toronto; X. O. Werder, of Pittsburg; H. W. Longyear and J. H. Carstens, of Detroit; W. B. Dorsett, of St. Louis.

Many interesting and valuable discussions took place on various phases of surgery of the kidney; on hemorrhage following celiotomy; on house-to-house operating; on surgery of the liver, gall-bladder and bile ducts; on the treatment of retrodisplacements of the uterus by the various methods, with a comparison of the results satisfactory, unsatisfactory, and dangerous to life. A complete report of the meeting will appear in the *American Journal of Obstetrics*, together with the papers read.

At the banquet held in the evening, Dr. Reamy, of Cincinnati, one of the contemporaries of Fordyce Barker, Emmet Thomas, H. P. C. Wilson, of Baltimore, and others who were the founders of the American Gynecological Society, a courteous gentleman, whose locks have become silvered with years, stated that as a guest of the Association he had listened with amazement to the discussions that had taken place, drinking in with pleasure all that he had heard. He said that, as a link with the past, his position was different to that of the others present; that he was able to compare the old and the new. He considered that the progress that had been made was marvelous and was glad to know that the progress had not yet ceased, but that the hand of the surgeon was still invading new fields for the relief of suffering humanity. The tribute to the work of the Association, in the twelfth year of its existence, coming from such a source and coming with all the freedom of a spontaneous utterance, without any attempt at flattery, must find a sympathetic echo in the breast of every Fellow of the Association, as it is but a recognition of patient toil.

One of the features of the meeting was the memorial address on the life and character of Lawson Tait, delivered by Dr. C. A. L. Reed, of Cincinnati. The Cincinnati *Enquirer* stated that Dr. Reed and Mr. Lawson Tait spent all night in the tower of the National Liberal Club in London, and quoted the last few sentences of a piece of very elegant diction. As the reporter of the *Enquirer* was pleased with this portion of the address, and as it is likely to please some of the readers of the *Practitioner* and show them that

Robert Louis Stevenson does not stand alone in writing rhyme in measured prose, we give it here :

"Mr. Tait was away from the hospital, away from the chamber of sickness, from the hall of controversy," said Dr. Reed, "and thus his great mind revelled in its freedom. England, the Indies, Africa, Anglo-American relations, the recent war between China and Japan, the contrasting features of the occidental and oriental civilisation, the great ethical movements of the world, music, the drama, human happiness, and life itself were themes that he touched with the spark of illumination. As we walked, Big Ben, in Parliament towei hard by, tolled twelve, anon one, and again two. The moon rose and the silhouette of the great city was seen against the tinted sky of the east. The speaker of great thoughts paused to view the majestic scene. I receded a step or two that I might contemplate, in clearer perspective, the more impressive picture of triumphant genius, with the world sleeping at his feet. Thus may he abide in peaceful memory !

Another feature of the meeting was the presence of Dr. W. Japp Sinclair, of Manchester, Eng., professor of obstetrics and gynecology in Owen's College, of the Victoria University. Dr. Sinclair was president of the section of obstetrics and gynecology at the recent meeting of the British Medical Association held in Montreal. As a guest of the Association he took part in the discussion. Owing to lack of time he will, unfortunately, not be able to visit Canada on this occasion.

The medical piofession of Indianapolis tendered a reception to the Association on Tuesday evening, to which all the Fellows and guests were cordially invited. Their courtesy was very highly appreciated by the Fellows.

The next meeting of the Association will take place in Louisville, Ky., under the presidency of Dr. Rufus B. Hall, of Cincinnati.

